# Advances in the Diagnosis and Management of Acute Coronary Syndrome: A Clinical Review

**DOI:** 10.7759/cureus.110344

**Published:** 2026-06-06

**Authors:** Anand Sekar G, Durga Prasad Rai, Bhagyesh Darji, Nikhilesh Jain, Saurabh Kumar, Richa Singh

**Affiliations:** 1 Department of Cardiology, Aarupadai Veedu Medical College and Hospital, Vinayaka Mission’s Research Foundation (Deemed to be University), Puducherry, IND; 2 Department of Cardiology, Sir Thutob Namgyal Memorial (STNM) Hospital, Gangtok, IND; 3 Department of Otorhinolaryngology (ENT), Dr. N. D. Desai Faculty of Medical Science and Research, Dharmsinh Desai University, Nadiad, IND; 4 Department of Critical Care Services, CARE Convenient Hospitals Ltd. (CHL) Hospitals, Indore, IND; 5 Department of Management, ARNI University, Kangra, IND; 6 School of Biotechnology, Jawaharlal Nehru University, New Delhi, IND

**Keywords:** acute coronary syndrome, biomarkers, imaging, management, precision medicine

## Abstract

Acute coronary syndrome (ACS) encompasses a spectrum of ischemic cardiac conditions associated with significant global morbidity and mortality, commonly driven by atherosclerotic plaque disruption and thrombosis, and remains a major clinical challenge despite advances in cardiovascular care. Persistent variability in early diagnosis, risk stratification, and the integration of emerging therapies into routine practice highlights existing gaps in translating evolving evidence into consistently improved clinical outcomes. This narrative clinical review aims to synthesise recent advances in diagnostic approaches and therapeutic strategies, focusing on biomarkers, imaging modalities, pharmacological interventions, and precision-based management. A structured narrative literature search was conducted using PubMed and Google Scholar for English-language human studies published between 2015 and 2025. Search terms included “acute coronary syndrome,” “high-sensitivity troponin,” “biomarkers,” “coronary computed tomography angiography,” “intravascular ultrasound,” “optical coherence tomography,” “antiplatelet therapy,” “anticoagulation,” “lipid-lowering therapy,” and “precision medicine.” Guidelines, randomised trials, systematic reviews, meta-analyses, and high-impact reviews were included, while duplicate, non-English, non-coronary, and clinically irrelevant studies were excluded. Study relevance and quality were narratively assessed using the Scale for the Assessment of Narrative Review Articles (SANRA) principles, with emphasis on justification of the manuscript’s importance, clarity of review aims, appropriateness of the literature search, balanced evidence presentation, scientific reasoning, and clinical relevance of the synthesis. Findings indicate that high-sensitivity troponins, advanced imaging techniques, and contemporary antithrombotic and lipid-lowering therapies have enhanced diagnostic accuracy and improved clinical outcomes. Emerging anti-inflammatory therapies and digital health innovations further contribute to more individualised patient management. These developments support the integration of novel tools with established clinical pathways to optimise care delivery. Continued research and improved implementation strategies remain necessary to address disparities and refine management approaches. A multidimensional, patient-centred framework is essential for advancing outcomes and guiding future clinical practice in ACS.

## Introduction and background

Acute coronary syndrome (ACS) includes ST-segment elevation myocardial infarction, non-ST-segment elevation myocardial infarction, and unstable angina. These conditions occur when coronary blood flow is suddenly reduced, leading to myocardial ischemia or necrosis, depending on the severity and duration of vessel obstruction [1,2]. ACS remains a major cause of cardiovascular morbidity and mortality worldwide. Its burden is unevenly distributed, with ACS-related age-standardised mortality remaining highest in lower-income regions. High-income countries have achieved more than 50% reductions in ACS mortality over the past two decades, compared with less than 15% reductions in lower-middle-income countries [1,3]. In many low- and middle-income settings, rising hospital presentations are further driven by population ageing, urbanisation, hypertension, diabetes, dyslipidemia, smoking, and unequal access to preventive cardiology [2].

The pathophysiology of ACS is heterogeneous. Plaque rupture is a major mechanism and typically involves disruption of a lipid-rich atherosclerotic plaque, platelet activation, thrombus formation, and partial or complete coronary occlusion [3,4]. Plaque erosion is now recognised as a distinct mechanism, particularly in some patients with non-obstructive or less severely stenotic lesions, and may present with different pathological features, clinical phenotypes, and treatment implications [5-7]. This distinction is clinically important because lesion morphology may influence diagnostic interpretation, invasive management, and the potential use of individualised treatment strategies.

Lipid deposition, endothelial dysfunction, inflammation, and thrombogenicity interact in the development and progression of ACS [4]. Biomarkers of myocardial injury, plaque instability, and thrombus formation have therefore become important for early diagnosis and risk prediction [4]. Inflammation contributes to endothelial dysfunction, plaque destabilisation, fibrous cap thinning, and thrombus formation, supporting the concept of ACS as an inflammatory-thrombotic as well as obstructive disease process [6,8]. Landmark inflammatory pathway studies, including CANTOS, COLCOT, and LoDoCo2, further support the clinical relevance of targeting inflammation in coronary artery disease and post-myocardial infarction care. Inflammatory mechanisms should therefore be considered alongside ischemic burden, plaque morphology, and thrombotic risk when refining ACS diagnosis and treatment [9].

Important knowledge gaps remain. Region-specific incidence data are still inconsistent, particularly in low- and middle-income countries. The contribution of plaque erosion, non-obstructive coronary lesions, and atypical presentations is not fully captured by conventional diagnostic pathways [7,9]. In addition, the implementation of high-sensitivity biomarkers, advanced imaging, structured risk scores, and guideline-directed therapies remains uneven across healthcare systems [10]. These gaps justify a contemporary review that integrates ACS epidemiology, pathophysiology, diagnostic innovation, and emerging management strategies to support earlier diagnosis, more precise treatment, and improved global outcomes [1].

Objectives of the review

This review aims to synthesise current evidence on integrated ACS management, with emphasis on diagnostic advances, therapeutic innovations, and precision-based clinical decision-making. Rather than addressing biomarkers, imaging, antithrombotic therapy, or lipid-lowering treatment as isolated topics, this review integrates these domains into a clinically oriented framework. It specifically evaluates high-sensitivity cardiac troponins, risk stratification tools, coronary and intravascular imaging, contemporary antithrombotic and lipid-lowering therapies, anti-inflammatory strategies, and digital health applications. The objective is to clarify how these advances can be combined within real-world ACS pathways to improve early diagnosis, individualised treatment selection, and long-term outcomes while identifying persistent gaps in implementation, access, patient heterogeneity, and evidence translation.

Literature search strategy

A structured narrative literature search was conducted using PubMed and Google Scholar to identify relevant English-language human studies published between 2015 and 2025. Search terms included “acute coronary syndrome,” “high-sensitivity troponin,” “biomarkers,” “coronary computed tomography angiography,” “intravascular ultrasound,” “optical coherence tomography,” “antiplatelet therapy,” “anticoagulation,” “lipid-lowering therapy,” “precision medicine,” and “artificial intelligence.” Guidelines, randomised controlled trials, systematic reviews, meta-analyses, and high-impact narrative reviews were prioritised. Studies were included if they addressed ACS epidemiology, pathophysiology, diagnosis, risk stratification, imaging, pharmacological management, interventional strategies, or emerging precision-based care. Duplicate articles, non-English studies, non-human studies, articles unrelated to ACS, and studies without clear clinical relevance were excluded. Titles, abstracts, and full texts were screened for relevance to the objectives of the review. Because this manuscript is a narrative clinical review rather than a systematic review, formal PRISMA reporting, quantitative meta-analysis, and formal risk-of-bias scoring were not performed. Studies were selected based on relevance, methodological quality, guideline importance, and clinical applicability to contemporary ACS management. When multiple sources addressed the same topic, priority was given to recent guideline documents, randomised controlled trials, large prospective studies, systematic reviews, meta-analyses, and studies with direct relevance to ACS diagnosis, management, or clinical outcomes. Study relevance and reporting quality were assessed narratively using Scale for the Assessment of Narrative Review Articles (SANRA) principles. Evidence was synthesised descriptively without pooled quantitative analysis; therefore, effect-size pooling, heterogeneity testing, publication-bias assessment, P-values, and confidence-interval reporting were not applicable.

## Review

High-sensitivity cardiac troponins in ACS

High-sensitivity cardiac troponins have transformed ACS diagnosis by enabling earlier and more accurate detection of myocardial injury, supporting faster emergency decision-making [11]. Rapid rule-in and rule-out pathways, particularly the guideline-supported 0/1-hour algorithm, have improved the early identification or exclusion of myocardial infarction and may reduce unnecessary hospitalization while improving resource use [12]. Clinical trials have demonstrated that faster troponin-based implementation strategies improve diagnostic efficiency without compromising patient safety and allow timely therapeutic intervention in high-risk patients presenting with chest pain [13].

The increased sensitivity of these assays may reduce specificity in patients with chronic kidney disease, heart failure, sepsis, structural heart disease, or advanced age because baseline troponin elevation may occur without acute myocardial infarction [3]. Therefore, diagnosis should not rely on a single elevated troponin value. Serial measurements, absolute or relative troponin changes, clinical presentation, electrocardiographic findings, renal function, and imaging results should be interpreted together to distinguish acute myocardial infarction from chronic myocardial injury and avoid unnecessary invasive procedures or overtreatment.

Combining high-sensitivity troponins with clinical risk scores, early assessment tools, advanced imaging, and novel biomarkers may further improve diagnostic pathways, prognostic assessment, and patient stratification in suspected ACS [7,14]. These findings support the central role of high-sensitivity cardiac troponins in contemporary ACS diagnosis while highlighting the need for optimized algorithms that remain applicable across diverse healthcare settings [11].

Risk stratification in ACS

Risk stratification is a central component of ACS evaluation because it identifies patients at higher risk of adverse cardiac events and guides diagnostic intensity, invasive management, and treatment decisions [15]. Thrombolysis in Myocardial Infarction (TIMI), Global Registry of Acute Coronary Events (GRACE), History, Electrocardiogram, Age, Risk Factors, and Troponin (HEART), and Emergency Department Assessment of Chest Pain Score (EDACS) scores have been widely studied in suspected ACS and acute chest pain populations, but they serve slightly different clinical purposes [16]. GRACE provides strong prognostic accuracy for mortality prediction, although it requires several clinical and laboratory variables. HEART and EDACS are more practical for emergency department chest pain assessment because they support early identification of low-risk patients who may be suitable for accelerated diagnostic pathways or discharge. TIMI is simple to apply but may have lower discriminatory performance in some emergency department populations [16,17].

Score selection should therefore depend on the clinical setting, available data, and the intended decision point, such as rapid emergency department disposition versus detailed prognostic assessment. The HEART pathway has demonstrated high sensitivity for identifying patients at low risk of 30-day major adverse cardiac events, commonly reported at approximately 96-100%, with moderate specificity, making it useful when combined with serial troponin testing and clinical judgment [17].

Combining clinical risk scores with biomarker data, particularly high-sensitivity troponin, can improve diagnostic and prognostic accuracy and support more individualized patient management in acute care settings [18]. Integration of risk stratification tools into clinical workflows may facilitate early discharge of low-risk patients and timely invasive evaluation of high-risk patients [19]. However, age, comorbidities, atypical presentations, and population-level variability limit the universal application of these models; therefore, formal scoring should complement rather than replace clinical judgment [20,21].

Machine learning and other advanced analytics are being investigated to improve risk prediction beyond conventional scoring systems. Reported models have incorporated clinical variables, high-sensitivity troponin kinetics, electrocardiographic features, coronary CT angiography findings, and intravascular imaging data to estimate myocardial infarction, recurrent ischemic events, and short-term major adverse cardiac events [22,23]. Although these tools may support more dynamic and individualized risk assessment, current evidence remains insufficient for routine standalone clinical use because many models still require prospective external validation, transparent reporting, workflow testing, and evaluation across diverse ACS populations. Overall, optimal risk stratification remains a key link between diagnosis and treatment because it supports evidence-based decision-making and improves clinical outcomes in patients with ACS within multidisciplinary care settings [24,25]. Table 1 shows the risk stratification tools used in ACS.

**Table 1:** Risk stratification tools in acute coronary syndrome. TIMI: Thrombolysis in Myocardial Infarction; GRACE: Global Registry of Acute Coronary Events; HEART: History, Electrocardiogram, Age, Risk factors, and Troponin; ACS: Acute coronary syndrome; MACE: Major adverse cardiovascular events; MI: Myocardial infarction; PRAISE: Prediction of Adverse Events Following an Acute Coronary Syndrome.

Score	Parameters Included	Clinical Use	Strength	Reference
TIMI	Age, risk factors, ECG changes	Predicts short-term risk	Simple and quick	[15]
GRACE	Age, vitals, creatinine, ECG	Mortality prediction	High prognostic accuracy	[17]
HEART	History, ECG, age, risk factors, troponin	ED risk stratification	Easy bedside use	[16]
Combined Scores	Risk score + troponin	Improved prediction accuracy	Better decision support	[18]
Validated AI-based Models	Clinical variables, ECG features, high-sensitivity troponin kinetics, imaging data	Prediction of ACS diagnosis, 30-day MACE, 1-year MACE, mortality, recurrent MI, and major bleeding	Examples include real-time ED AI models for chest pain cohorts, ECG-based machine-learning models for acute chest pain mortality risk, and PRAISE for 1-year post-ACS mortality, recurrent MI, and bleeding prediction	[25]

Imaging modalities in ACS

Cardiovascular imaging has become an established component of ACS evaluation, particularly when symptoms, electrocardiography, and biomarkers provide inconclusive results [19]. Coronary computed tomography angiography (CCTA) is an established noninvasive modality in many contemporary emergency and chest pain pathways rather than an entirely emerging technique. In low- to intermediate-risk patients with acute chest pain, CCTA enables rapid visualization of coronary anatomy and has high sensitivity and negative predictive value for excluding clinically significant coronary artery disease. Recent reviews report CCTA sensitivity of approximately 90-100% and negative predictive value of 98-100% for ruling out significant coronary artery disease in selected acute chest pain populations [20,21].

The ISCHEMIA trial also has indirect relevance to ACS imaging because it demonstrated the value of anatomic imaging in refining patient selection before invasive management, although the trial primarily enrolled patients with stable ischemic heart disease rather than acute ACS. In ISCHEMIA, CCTA was used to exclude left main disease and non-obstructive coronary artery disease before randomization, reinforcing the role of coronary anatomy in guiding downstream treatment decisions. In suspected ACS, combining high-sensitivity troponin testing with CCTA may improve early diagnostic confidence by integrating biochemical evidence of myocardial injury with anatomical assessment of coronary disease [22].

Intracoronary imaging, including intravascular ultrasound (IVUS) and optical coherence tomography (OCT), provides detailed assessment of plaque morphology, thrombus burden, plaque rupture, plaque erosion, lesion severity, stent expansion, malapposition, and edge dissection [4,5]. These features can influence real-world decision-making by helping clinicians identify the culprit lesion, select stent size and length, optimize stent deployment, and determine whether additional post-dilatation or adjunctive pharmacotherapy is required. Evidence from ILUMIEN III showed that OCT-guided PCI produced stent expansion comparable to IVUS-guided PCI and improved procedural assessment compared with angiography alone, while ILUMIEN IV showed a larger post-PCI minimum stent area with OCT guidance, although improvement in the primary clinical endpoint of target-vessel failure was not clearly demonstrated. These findings suggest that intracoronary imaging improves procedural optimization, but its effect on hard clinical outcomes may depend on patient selection, lesion complexity, operator expertise, and institutional workflow.

The clinical utility of OCT and IVUS is also influenced by real-world resource availability. In centers with limited intracoronary imaging expertise, routine use may be restricted by cost, procedure time, catheter availability, contrast load, and the need for trained operators. Selective use may therefore be most appropriate in complex lesions, ambiguous culprit lesions, left main disease, stent failure, suspected plaque erosion, or cases where angiography alone does not adequately explain the clinical presentation. Training programs, standardized imaging protocols, tele-imaging support, and integration with automated interpretation tools may improve adoption in lower-resource settings.

Artificial intelligence-assisted plaque characterization is being investigated as an extension of cardiovascular imaging rather than as a fully established ACS decision-making tool. Current applications include automated plaque segmentation and high-risk plaque detection using CCTA, OCT, and IVUS data, along with CCTA-based platforms, such as Cleerly and HeartFlow Plaque Analysis, that quantify stenosis, plaque burden, plaque composition, and high-risk features. These tools may improve reproducibility and workflow efficiency, but their clinical use in ACS pathways should remain cautious until external validation, cost-effectiveness analyses, prospective workflow testing, and outcome-based evidence confirm that AI-guided plaque assessment improves clinical decision-making.

Intravascular imaging and plaque characterization

Intravascular imaging provides high-resolution assessment of coronary plaque and vessel morphology beyond conventional angiography [23]. OCT and IVUS help identify plaque characteristics such as fibrous cap thickness, lipid core size, thrombus burden, plaque rupture, and plaque erosion [24]. OCT provides higher spatial resolution, usually around 10-20 µm, making it particularly useful for detecting thin-cap fibroatheroma, small thrombi, plaque erosion, and stent malapposition. IVUS has lower spatial resolution, usually around 100-150 µm, but provides deeper tissue penetration, making it more useful for assessing vessel size, plaque burden, remodeling, and left main or large-vessel disease. Therefore, OCT and IVUS are complementary rather than interchangeable modalities in ACS evaluation and PCI optimization. By characterizing vulnerable plaque and procedural anatomy, these modalities can support culprit-lesion identification, stent sizing, stent optimization, and selection of adjunctive pharmacotherapy [25]. Intracoronary imaging also complements PCI by improving lesion assessment, confirming adequate stent expansion, and potentially reducing the risk of complications such as stent thrombosis and restenosis [26].

These modalities can support lesion-level assessment and help align PCI planning with the underlying plaque mechanism [6]. Their routine use, however, may be limited by cost, procedural complexity, catheter availability, contrast requirements, and the need for trained operators, particularly in resource-limited settings [2]. Emerging innovations, including artificial intelligence-assisted plaque analysis and automated multimodal image integration, may improve diagnostic consistency, but broader validation is required before routine clinical adoption [8]. Overall, intravascular imaging helps bridge anatomical assessment and functional decision-making in ACS, particularly when angiography alone does not adequately define plaque mechanism, lesion severity, or PCI optimization needs [1]. Table 2 shows selected significant diagnostic modalities used in ACS.

**Table 2:** Diagnostic modalities in acute coronary syndrome. MI: Myocardial infarction; STEMI: ST-elevation myocardial infarction; NSTEMI: Non-ST-elevation myocardial infarction; CAD: Coronary artery disease; IVUS: Intravascular ultrasound; OCT: Optical coherence tomography.

Modality	Principle	Clinical Utility	Limitation	Reference
High-sensitivity troponin	Detects myocardial injury biomarkers	Early diagnosis and rule-in/rule-out of MI	False positives in comorbidities	[11]
ECG	Electrical activity of the heart	Rapid initial assessment and STEMI identification	Low sensitivity in NSTEMI	[3]
Coronary CT angiography	Noninvasive coronary visualization	Excludes CAD in low-risk patients	Radiation exposure and contrast use	[20]
IVUS	Ultrasound-based intravascular imaging	Plaque burden and vessel structure	Operator-dependent	[24]
OCT	High-resolution optical imaging	Plaque characterization and thrombus detection	Limited penetration depth	[23]

Guideline-based management of ACS

Evidence-based guidelines from major cardiovascular societies provide the framework for contemporary ACS management by integrating randomized trial evidence, observational data, and expert consensus into standardized care pathways [27]. The 2023 ESC guidelines emphasize early risk stratification, rapid initiation of antithrombotic therapy, and an invasive strategy based on ischemic and bleeding risk, with immediate reperfusion for STEMI and immediate or early invasive evaluation for high-risk NSTE-ACS [28]. ACC/AHA guideline updates similarly support prompt reperfusion, dual antiplatelet therapy, anticoagulation, high-intensity lipid-lowering therapy, and risk-guided invasive management, but differ in some practical recommendations regarding timing, antithrombotic selection, and regional implementation pathways [28]. Contemporary guideline frameworks also emphasize patient-centered decision-making by considering comorbidities, bleeding risk, frailty, socioeconomic factors, and local resource availability [29].

The occlusive myocardial infarction paradigm has been proposed to complement traditional STEMI and NSTEMI categories by identifying patients who may benefit from reperfusion despite the absence of classic ST-segment elevation [30]. This evolving approach reinforces the need to integrate high-sensitivity troponin testing, electrocardiographic interpretation, clinical risk assessment, and imaging when evaluating suspected ACS [12]. Guideline implementation remains variable because of differences in healthcare infrastructure, clinician expertise, patient characteristics, and access to diagnostic or interventional resources [9].

**Figure 1:**
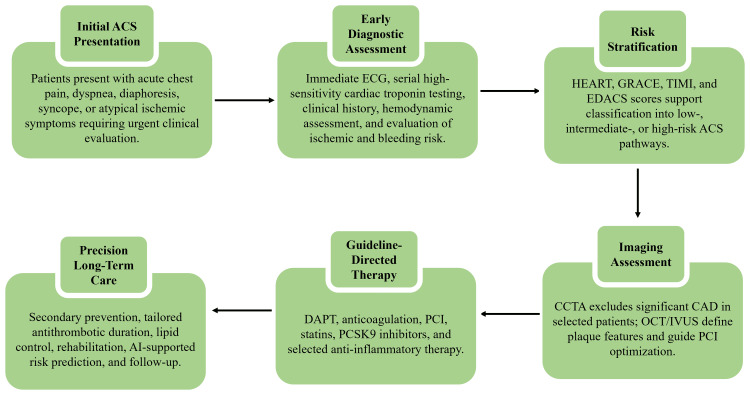
Integrated ACS diagnostic and management pathway. Created by the authors using Microsoft PowerPoint. ACS: Acute coronary syndrome; hs-troponin: High-sensitivity cardiac troponin; HEART: History, Electrocardiogram, Age, Risk factors, and Troponin; GRACE: Global Registry of Acute Coronary Events; TIMI: Thrombolysis in Myocardial Infarction; EDACS: Emergency Department Assessment of Chest Pain Score; CCTA: Coronary computed tomography angiography; CAD: Coronary artery disease; OCT: Optical coherence tomography; IVUS: Intravascular ultrasound; PCI: Percutaneous coronary intervention; DAPT: Dual antiplatelet therapy; PCSK9: Proprotein convertase subtilisin/kexin type 9.

Continuing medical education, quality improvement programs, and digital decision-support tools may help improve adherence to guideline-based ACS care and reduce variation in clinical practice [8]. Guidelines provide a structured basis for systematic clinical decision-making while allowing adaptation to individual patient risk, comorbidities, and resource context [1]. Figure 1 shows the integrated ACS pathway from diagnosis and risk stratification to imaging, treatment, and long-term care.

Antiplatelet therapy in ACS

Antiplatelet therapy is central to ACS management because platelet activation and aggregation drive thrombus formation after plaque disruption and vascular injury [3]. DAPT with aspirin and a P2Y12 receptor inhibitor is the standard antiplatelet approach in ACS, with ticagrelor and prasugrel generally providing stronger platelet inhibition than clopidogrel in appropriate patients. Agent selection should be individualized according to ACS type, planned invasive strategy, ischemic risk, bleeding risk, comorbidities, and contraindications. Prasugrel is generally preferred in selected PCI-treated ACS patients without high bleeding risk, but it is contraindicated in patients with previous stroke or transient ischemic attack and should be avoided or used cautiously in patients aged ≥75 years or weighing <60 kg. Ticagrelor may be used in a broader range of ACS patients, including those managed invasively or medically, but dyspnea, bradyarrhythmia risk, drug interactions, adherence, and bleeding risk should be considered [31].

Comparative evidence supports the use of potent P2Y12 inhibitors to reduce recurrent ischemic events in selected ACS patients, although bleeding risk remains a key determinant of treatment choice [32]. Other strategies, such as early aspirin discontinuation with continuation of P2Y12 inhibitor monotherapy, have recently been investigated and may reduce bleeding complications without significantly increasing ischemic events in selected patient populations [33]. This approach may be particularly beneficial in patients with high bleeding risk, elderly individuals, patients requiring long-term anticoagulation, those with prior gastrointestinal bleeding, or clinically stable patients after successful PCI with low residual ischemic risk. Careful patient selection and individualized assessment of ischemic versus bleeding risk remain essential before adopting shortened DAPT strategies.

DAPT duration should be individualized according to ischemic risk, bleeding risk, procedural features, comorbidities, and long-term treatment goals [34]. Antiplatelet therapy remains a core component of ACS management, but optimization can be challenging in patients with high bleeding risk, polypharmacy, drug intolerance, or contraindications [2,6].

Recent research has focused on optimizing antiplatelet therapy through pharmacogenomic and precision-based approaches. For example, CYP2C19 loss-of-function alleles are associated with reduced clopidogrel activation and a higher risk of ischemic events, supporting genotype-guided selection of ticagrelor or prasugrel in selected ACS patients. Platelet function testing may also help identify high on-treatment platelet reactivity or excessive platelet inhibition, allowing escalation or de-escalation of therapy according to ischemic and bleeding risk. These approaches may be most useful in patients with recurrent ischemic events, high bleeding risk, planned long-term antiplatelet therapy, or uncertain response to clopidogrel.

Anticoagulation strategies in ACS

Anticoagulant therapy is used with antiplatelet therapy in ACS to inhibit activation of the coagulation cascade and limit thrombus propagation after plaque disruption [6]. Unfractionated heparin and low-molecular-weight heparin remain established anticoagulants in both invasive and conservative ACS strategies and reduce ischemic complications when used appropriately in the acute setting [2]. DOACs should not be considered routine anticoagulants for all ACS patients. Their use remains limited to selected clinical contexts, particularly in patients with ACS who also require long-term oral anticoagulation for atrial fibrillation, venous thromboembolism, or other established indications [35]. In these patients, DOAC-based regimens may reduce the need for vitamin K antagonist therapy and simplify anticoagulation, but the balance between ischemic protection and bleeding risk must be carefully assessed [36]. Bleeding risk assessment using tools such as HAS-BLED and PRECISE-DAPT may help guide antithrombotic intensity, DAPT duration, and decisions regarding dual versus triple therapy in patients requiring combined antithrombotic treatment.

**Figure 2:**
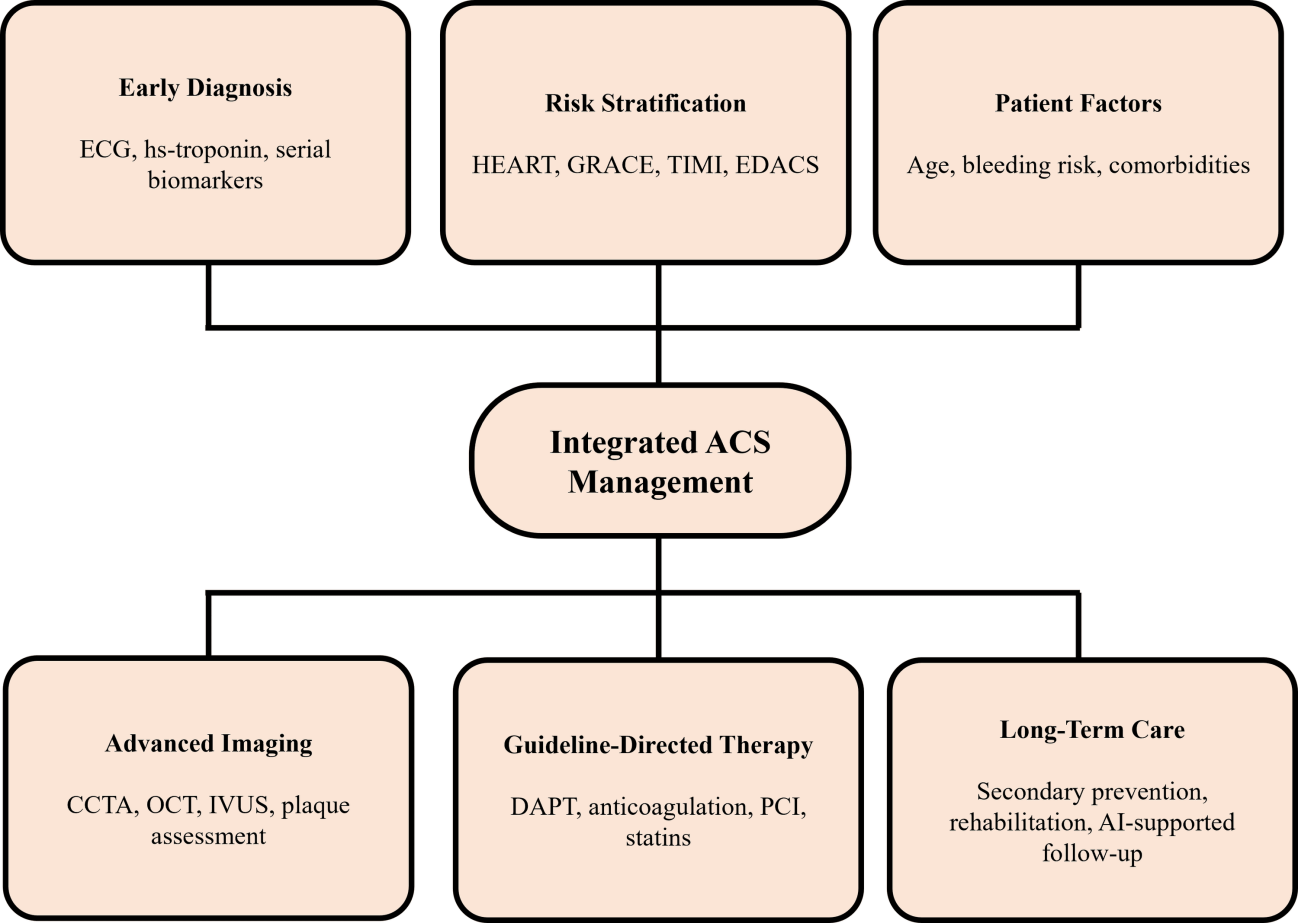
Integrated ACS management framework. Created by the authors using Microsoft PowerPoint. ACS: Acute coronary syndrome; hs-troponin: High-sensitivity cardiac troponin; HEART: History, Electrocardiogram, Age, Risk factors, and Troponin; GRACE: Global Registry of Acute Coronary Events; TIMI: Thrombolysis in Myocardial Infarction; EDACS: Emergency Department Assessment of Chest Pain Score; CCTA: coronary computed tomography angiography; CAD: coronary artery disease; OCT: optical coherence tomography; IVUS: intravascular ultrasound; PCI: percutaneous coronary intervention; DAPT: dual antiplatelet therapy.

DOAC use in complex ACS populations requires attention to drug interactions, adherence, renal function, and heterogeneity in clinical outcomes across patient groups [37]. Real-world evidence can support the selective use of these agents, but treatment decisions should remain individualized according to thrombotic risk, bleeding risk, comorbidities, renal function, and the need for concomitant antiplatelet therapy [38]. Overall, anticoagulation in ACS requires careful balancing of ischemic protection and bleeding risk, particularly in patients with renal impairment, multiple cardiovascular comorbidities, or indications for long-term oral anticoagulation [9]. Further research should refine individualized anticoagulation strategies and clarify how best to integrate anticoagulants with antiplatelet and interventional therapies to improve outcomes and reduce complications [1]. Figure 2 summarizes key diagnostic, risk-based, imaging, therapeutic, and long-term care components of ACS management.

Lipid-lowering therapy in ACS

Lipid-lowering therapy is a central component of secondary prevention after ACS because atherogenic lipoproteins contribute to coronary plaque progression, instability, and recurrent ischemic events [1]. Current guideline-based care generally recommends early high-intensity statin therapy with an LDL cholesterol target of <55 mg/dL and at least 50% LDL cholesterol reduction in very-high-risk ACS patients [38]. High-intensity statins typically reduce LDL cholesterol by approximately 50%, while ezetimibe provides an additional 15-25% reduction when added to statin therapy. PCSK9 inhibitors such as evolocumab and alirocumab can reduce LDL cholesterol by approximately 50-60% beyond background therapy and have shown cardiovascular outcome benefits in major trials such as FOURIER and ODYSSEY OUTCOMES [39-41]. Inclisiran lowers LDL cholesterol by approximately 50% through twice-yearly maintenance dosing, while bempedoic acid reduces LDL cholesterol by approximately 15-25% and may be useful in selected patients with statin intolerance or persistent LDL elevation [42].

Despite these therapeutic advances, global access remains uneven. High medication costs, limited insurance coverage, inconsistent availability of PCSK9-directed therapies, inadequate lipid monitoring, and poor long-term adherence restrict optimal lipid control, particularly in low- and middle-income countries. These disparities may delay LDL target achievement after ACS and contribute to preventable recurrent cardiovascular events. Table 3 shows the most important pharmacological management strategies in ACS.

**Table 3:** Pharmacological management in acute coronary syndrome. MI: Myocardial infarction; ACS: Acute coronary syndrome; UFH: Unfractionated heparin; LMWH: Low-molecular-weight heparin; DOAC: Direct oral anticoagulant; LDL: Low-density lipoprotein; PCSK9: Proprotein convertase subtilisin/kexin type 9; ASCVD: Atherosclerotic cardiovascular disease; ATP: Adenosine triphosphate.

Drug Class	Examples	Mechanism of Action	Major Cardiovascular Outcome Evidence	Reference
Antiplatelets	Aspirin, ticagrelor, prasugrel	Inhibit platelet activation and aggregation	Reduce recurrent MI, stent thrombosis, and ischemic events after ACS	[31-34]
Anticoagulants	UFH, LMWH, selected DOAC-based regimens	Inhibit thrombin generation or factor Xa activity	Reduce thrombus propagation and ischemic complications; DOAC use is limited to selected ACS patients with separate anticoagulation indications	[36-38]
Statins	Atorvastatin, rosuvastatin	Reduce LDL cholesterol and stabilize plaque	Reduce recurrent major adverse cardiovascular events and support plaque stabilization after ACS	[39]
PCSK9 inhibitors	Evolocumab, alirocumab	Increase LDL receptor recycling and LDL clearance	Reduce LDL cholesterol and recurrent cardiovascular events in very-high-risk ASCVD/ACS populations	[40,41]
Anti-inflammatory therapy	Colchicine	Inhibits inflammatory pathways, including inflammasome-mediated signaling	Reduces recurrent ischemic cardiovascular events in selected coronary artery disease and post-ACS populations	[43,44]
Additional lipid-lowering agents	Ezetimibe, inclisiran, bempedoic acid	Reduce intestinal cholesterol absorption, inhibit PCSK9 synthesis, or inhibit ATP-citrate lyase	Provide additional LDL reduction; ezetimibe and bempedoic acid have outcome-supporting evidence, while inclisiran has strong LDL-lowering evidence with ongoing outcomes evaluation	[42,45]

Future directions and precision medicine in ACS

Future ACS management should translate precision medicine from concept to routine clinical implementation. Real-world examples include genotype-guided antiplatelet selection using CYP2C19 testing to identify patients with reduced clopidogrel activation, bleeding-risk-guided shortening of DAPT using PRECISE-DAPT or ARC-HBR criteria, and LDL cholesterol target-based escalation from high-intensity statins to ezetimibe, PCSK9 inhibitors, inclisiran, or bempedoic acid in very-high-risk patients [46,47]. Biomarker-based pathways using serial high-sensitivity troponin algorithms can support faster rule-in and rule-out decisions in emergency departments, while imaging-guided PCI using OCT or IVUS can help optimize stent expansion, identify plaque rupture or erosion, and guide treatment in complex lesions [25,39].

Digital health and artificial intelligence may support ACS care by assisting ECG interpretation, CCTA plaque assessment, recurrent ischemic or bleeding risk prediction, specialist triage, remote cardiac rehabilitation, medication adherence monitoring, and symptom follow-up. However, important limitations remain [48]. Many AI models are developed using single-center or demographically narrow datasets, which limits generalizability across regions, ethnic groups, healthcare systems, and resource settings. Key validation concerns include dataset shift, limited external validation, inconsistent outcome definitions, insufficient prospective testing, and incomplete integration into real-time clinical workflows. Ethical concerns include algorithmic bias, data privacy, transparency, explainability, clinician accountability, and the risk of over-reliance on automated recommendations [49]. Therefore, AI-based ACS tools should be used as decision-support systems rather than replacements for clinical judgment, and their implementation should require prospective validation, auditability, equity assessment, and regulatory oversight [50].

**Figure 3:**
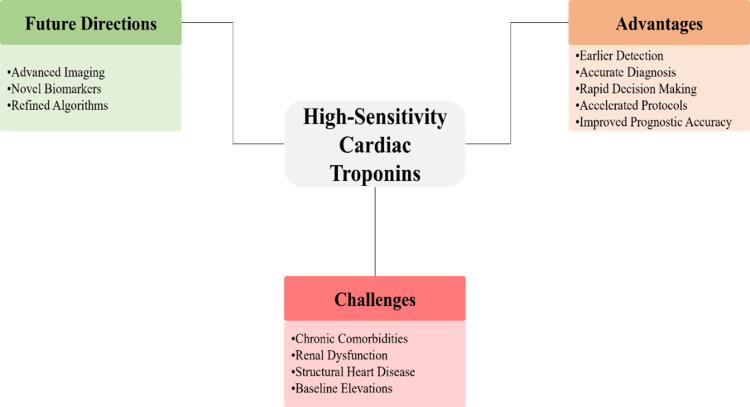
Role of high-sensitivity cardiac troponins in acute coronary syndrome diagnosis. Created by the authors using Microsoft PowerPoint.

Implementation also remains constrained by cost, data quality, interoperability, clinician training, algorithm transparency, and unequal access to advanced diagnostics and therapies, particularly in low-resource settings [2]. Future research should prioritize pragmatic trials, external validation, workflow integration, and equitable implementation rather than technology development alone. Figure 3 summarizes the role of high-sensitivity cardiac troponins in ACS diagnosis, including key advantages, limitations, and future opportunities.

Limitations and future directions

The available evidence on ACS is limited by heterogeneity in study populations, clinical presentations, diagnostic criteria, treatment protocols, and healthcare infrastructure across regions. As this is a narrative review, additional limitations include the possibility of selection bias, incomplete retrieval of relevant studies, lack of formal meta-analysis, and reliance on qualitative synthesis rather than pooled quantitative estimates. Most clinical trials are performed under controlled conditions that may not always reflect real-world practice, particularly in resource-limited settings.

In addition, variability in diagnostic criteria and treatment regimens makes comparisons across studies difficult, and the inadequate representation of older adults and women limits generalizability. This is clinically important because women with ACS more frequently present with atypical symptoms, delayed diagnosis, and differences in plaque morphology, including higher recognition of plaque erosion or non-obstructive disease. Older adults often have atypical presentations, multiple comorbidities, renal dysfunction, frailty, and baseline troponin elevation, which can complicate biomarker interpretation and risk stratification. Age- and sex-related differences may also influence treatment response, bleeding risk, antithrombotic selection, invasive management decisions, and tolerance of intensive lipid-lowering therapy.

Future research needs to focus on multicenter studies with large sample sizes that include diverse populations to enhance the external validity and applicability of the findings. Studies should also pay greater attention to precision medicine approaches, including genomic profiling and biomarker-based therapy, to individualize treatment strategies. AI and digital health solutions may allow better diagnosis and earlier risk prediction. Furthermore, improving guideline implementation and reducing global disparities in access to advanced diagnostic and treatment modalities remain necessary.

## Conclusions

ACS remains a major clinical challenge because timely diagnosis, accurate risk stratification, and individualized treatment must occur within narrow therapeutic windows. High-sensitivity troponins, structured risk scores, CCTA, OCT, IVUS, contemporary antithrombotic therapy, intensive lipid-lowering therapy, and selected anti-inflammatory strategies have improved diagnostic and therapeutic precision. In clinical practice, these tools should be applied through guideline-directed pathways that balance ischemic risk, bleeding risk, comorbidities, patient age, sex-specific considerations, resource availability, and long-term secondary prevention needs. Persistent gaps include unequal access to advanced diagnostics and therapies, underrepresentation of women and older adults, and limited real-world validation of AI-based and precision-medicine tools. Future progress should prioritize practical implementation, external validation, equitable access, and multidisciplinary care to reduce recurrent events and improve long-term outcomes after ACS.
